# Association of *NAT2* polymorphisms with risk of colorectal adenomas: Evidence from 3,197 cases and 4,681 controls

**DOI:** 10.3892/etm.2012.695

**Published:** 2012-09-04

**Authors:** WENLEI ZHUO, LIANG ZHANG, ZHIQUN QIU, LEI CAI, BO ZHU, ZHENGTANG CHEN

**Affiliations:** 1 Institute of Cancer, Xinqiao Hospital, Third Military Medical University;; 2 Department of Environmental Hygiene, College of Preventive Medicine, Third Military Medical University;; 3 Institute of Hepatobiliary Surgery, Southwest Hospital, Third Military Medical University, Chongqing, P.R. China

**Keywords:** *NAT2*, susceptibility, colorectal adenoma, meta-analysis, polymorphism

## Abstract

Previous studies have implicated *NAT2* polymorphisms as risk factors for various types of cancer. Colorectal adenomas are recognized as a pre-neoplastic lesion. A growing body of research documenting the association of *NAT2* polymorphisms with the risk of colorectal adenomas has yielded conflicting results. The aim of the present study was to derive a more precise estimation of this association. Meta-analyses assessing the association of *NAT2* variants with colorectal adenomas were conducted and subgroup analyses on smoking status and the source of the controls were also performed. Eligible studies were identified for the period before March 2012. A total of seven case-control studies, including 3,197 cases and 4,681 controls, were selected following extensive searching and screening. In the overall data, no associations between *NAT2* polymorphisms and colorectal adenomas were observed [odds ratio (OR), 1.04; 95% confidence interval (CI), 0.90–1.21]. However, in the subgroup analysis concerning smoking status, slow acetylator variants were revealed to be correlated with increased colorectal adenoma risk in individuals who have smoked (OR, 1.31; 95% CI, 1.04–1.64). In conclusion, the data of the present study suggested that *NAT2* polymorphisms may be a risk factor for colorectal adenomas in individuals who have a history of smoking.

## Introduction

Colorectal cancer is one of the most common malignancies worldwide, particularly in Western populations, and is thought to be correlated with colorectal adenoma, a type of pre-neoplastic lesion. The mechanisms of colon adenoma genesis are still unclear.

Evidence indicates that certain epidemiological factors, including cigarette smoking, alcohol use and meat consumption, may contribute to the risk of colorectal adenomas (1,2). Moreover, exposure to certain toxins, such as the heterocyclic aromatic amines formed during the cooking of meat at high temperatures, may increase colorectal adenoma risk (3). However, although numerous individuals are exposed to environmental risk factors, colorectal adenomas develop in only a small proportion of these individuals. Additionally, previous studies suggested a marked association between colonic adenoma risk and a family history of adenomas (4,5), indicating that host genetic factors may play a critical role in the genesis of colorectal adenomas.

Colon tissue is vulnerable to the effects of external toxins by direct exposure. Xenobiotics can be bio-activated into their ultimate carcinogen forms by phase I enzymes and subsequently detoxified by phase II enzymes, such as *CYP1A1* and *GSTM1,* respectively (6). Genetic variation in the genes encoding these enzymes may affect carcinogen activation/detoxification and modulate DNA repair, possibly by altering the genes’ expression and function. This may result in oncogenesis.

Acetylation is an important biotransformation route for these chemicals. In humans, the N-acetyltransferase 2 (*NAT2*) gene encodes a phase II enzyme that plays an essential role in the metabolism of aromatic heterocyclic amines and hydrazines via N- and O-acetylation (7). Alterations to the *NAT2* acetylator status caused by variations in the *NAT2* gene have been reported to reduce enzymatic activity, resulting in inefficient detoxification and thus leading to increased cancer susceptibility (8). Several *NAT2* genetic variants have been identified in humans, of which NAT2*4 is regarded as the most common allele linked to rapid acetylation. NAT2*12A, NAT2*12C, NAT2*13 and NAT2*18 have also been classified as rapid alleles. The remaining alleles are considered to be slow alleles (9–11).

Published studies have been conducted on the association of *NAT2* genetic variants with colorectal adenoma risk and have yielded inconclusive results. Whether *NAT2* polymorphisms are a risk factor for colorectal adenoma remains uncertain. Therefore, in the present study, evidence-based quantitative meta-analyses of the published studies were performed to derive a more precise estimation of this association.

## Materials and methods

### Literature search strategy

A search of the Medline, EMBASE, OVID, Sciencedirect, Google scholar and CNKI databases was performed covering all studies published before March 2012 with no language limitations. Combinations of the following keywords were used: *NAT2*, *Nacetyltransferase 2*, *colon*, *colorectal*, *neoplasm*, *polyp*, *adenoma* and *polymorphism*. The bibliographies of all the retrieved studies were searched for further relevant publications. Review articles and the bibliographies of other identified relevant studies were searched manually to identify additional eligible studies.

### Inclusion criteria

The following criteria were used for the literature selection: i) studies should be concerned with the association of *NAT2* polymorphisms with colorectal adenoma risk; ii) studies should be observational (case-control or cohort); iii) studies must present the sample size, odds ratios (ORs) and the corresponding 95% confidence intervals (CIs) and genetic distributions or information that may aid the interpretation of the results. After extensive searching, all studies were reviewed in accordance with the criteria defined above for further analysis.

### Data extraction

Data were extracted and entered into a database by two reviewers independently. In the case of conflicting evaluations, agreement was reached following discussion. If a consensus could not be reached, a third reviewer was consulted to resolve the dispute and a final decision was then made according to the majority decision. Carriers of at least one of the high-activity alleles were identified as rapid acetylators and individuals carrying two low-activity alleles were classified as slow acetylators, as stated in the primary literature.

### Statistical analysis

The OR of the *NAT2* polymorphisms and colorectal adenoma risk was estimated for each study. To detect possible sample size biases, the OR and corresponding 95% CI of each study were plotted against the number of participants. A Chi-square-based Q statistic test was performed to assess heterogeneity. If the result of the heterogeneity test was P>0.1, ORs were pooled according to the fixed-effects model (Mantel-Haenszel). Otherwise, the random-effects model (DerSimonian and Laird) was used. The significance of the pooled ORs was determined using a Z-test.

Publication bias was assessed by visual inspection of funnel plots (12) in which the standard error of the log(OR) of each study was plotted against the corresponding log(OR). An asymmetric plot indicates a possible publication bias. The symmetry of the funnel plot was evaluated using Egger’s linear regression test (13). Statistical analyses were performed using the STATA 11.0 software (StataCorp, College Station, TX, USA).

## Results

### Literature search and meta-analysis databases

**Figure 1:**
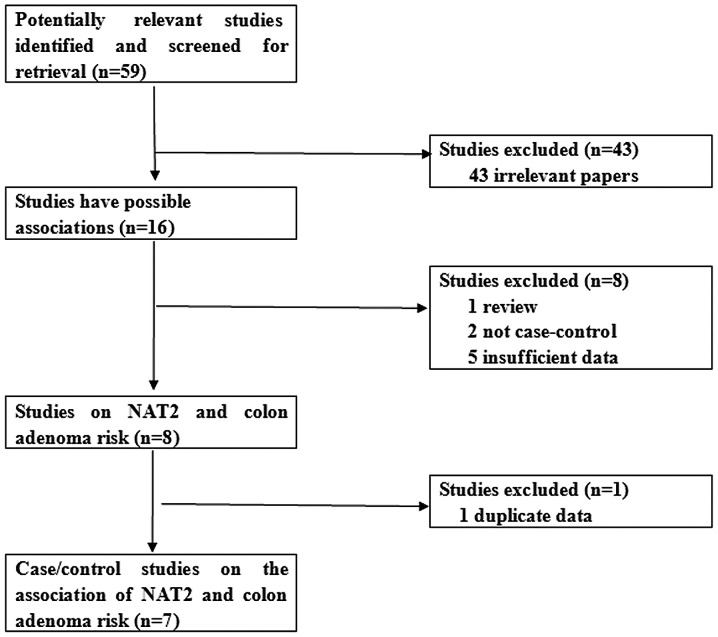
Flow diagram of included/excluded studies in this meta-analysis. NAT2, N-acetyltransferase 2.

As shown in Fig. 1, a total of 59 publications were searched and screened for retrieval, of which 43 irrelevant studies were excluded. Thus, 16 studies were primarily identified, of which one review was excluded (14). Subsequently, two articles which were not case-control studies (15,16) and five studies lacking sufficient information (17–21) were also excluded. Eight publications were identified (22–29). Two studies conducted by Tiemersma *et al* (22,28) concerned the same research subjects. Thus, the study concerning cigarette smoking was selected (28). Finally, seven case-control studies were selected (23–29).

The included studies were all written in English, of which one involved a Caucasian population (28) and the remaining six involved multi-ethnic populations. We were able to extract information about smoking status from two of the studies (25,28).

We established a database of the extracted information from each study. The relevant information is shown in Table I. The first author and the number and characteristics of cases and controls for each study are presented, as well as the other necessary information. The genetic distributions of the control groups were in Hardy-Weinberg equilibrium. The distributions of the *NAT2* acetylator variants (classified as rapid or slow) are presented in Table II.

### Test of heterogeneity

As shown in Table III, the heterogeneity of the overall data was significant since the P-value of the Q tests was <0.1 and thus the random-effects model was used. However, when subgroup analysis with regard to the source of the controls was conducted, no heterogeneity in the hospital-based subgroup was found.

### Quantitative data synthesis

**Figure 2:**
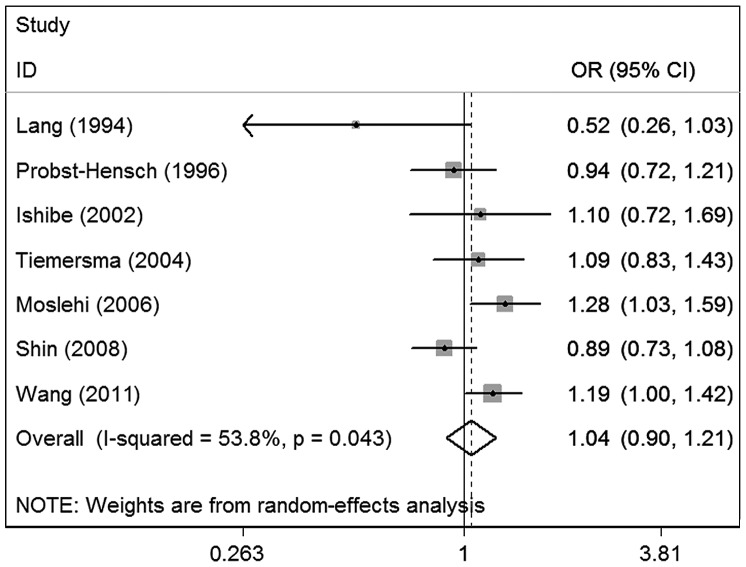
Meta-analysis of the association of colorectal adenoma risk with *NAT2* polymorphisms for the overall data (slow vs. rapid). OR, odds ratio; 95% CI, 95% confidence interval; NAT2, N-acetyltransferase 2.

Table III lists the main results of the meta-analysis. The overall data from the seven studies containing 3,197 cases and 4,681 controls revealed no significant associations between *NAT2* polymorphisms and colorectal adenoma risk (OR, 1.04; 95% CI, 0.90–1.21; P=0.043 for heterogeneity), suggesting that *NAT2* polymorphisms may have little association with colorectal adenoma risk (Fig. 2).

**Figure 3:**
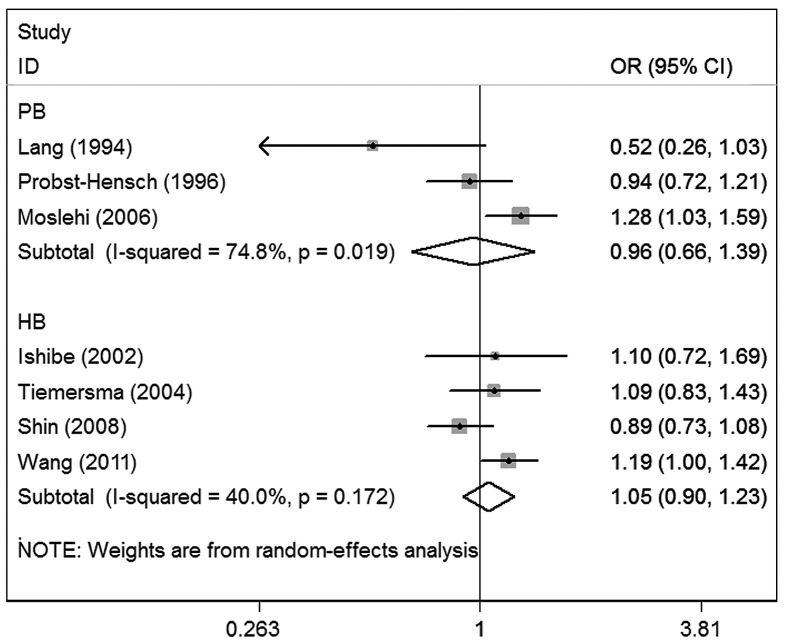
Meta-analysis of the association of colorectal adenoma risk with *NAT2* polymorphisms (slow vs. rapid; stratified by source of controls). HB, hospital-based; PB, population-based; OR, odds ratio; 95% CI, 95% confidence interval; NAT2, N-acetyltransferase 2.

**Figure 4:**
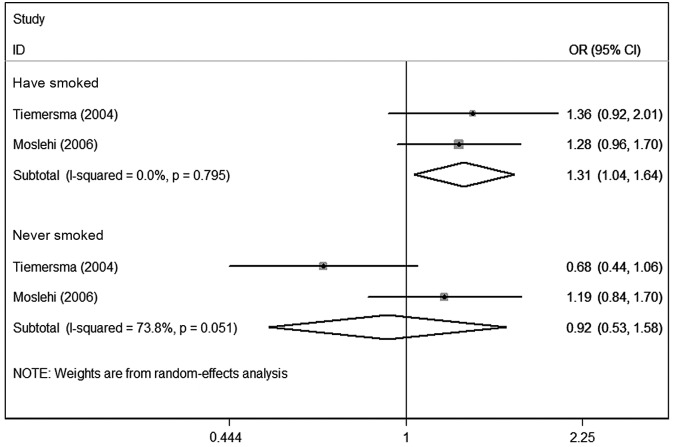
Meta-analysis of the association of colorectal adenoma risk with *NAT2* polymorphisms (slow vs. rapid; stratified by smoking status). OR, odds ratio; 95% CI, 95% confidence interval; NAT2, N-acetyltransferase 2.

To further assess the possible impact of smoking and the source of the controls on the results, relevant data were extracted to conduct subgroup analyses. In the subgroup analysis of the source of the controls, no significant associations were observed in either the hospital-based group (OR, 1.05; 95% CI, 0.90–1.23; P=0.172 for heterogeneity) or the population-based group (OR, 0.96; 95% CI, 0.66–1.39; P=0.019 for heterogeneity; Fig. 3). In the smoking status subgroups, the data showed that slow acetylator variants may be associated with increased colonic adenoma risk in smokers (OR, 1.31; 95% CI, 1.04–1.64; P=0.795 for heterogeneity). Nevertheless, no marked associations were observed in the never smoked subgroup (OR, 0.92; 95% CI, 0.53–1.58; P=0.051 for heterogeneity; Fig. 4).

An attempt was made to extract relevant data concerning meat and alcohol consumption to use in the subgroup analysis, but insufficient data were available.

### Sensitivity analysis

In order to compare the differences and evaluate the sensitivity of the meta-analyses, the results of the fixed-effects model for the overall data were also reported. The combined OR and 95% CI were 1.07 and 0.97–1.17, respectively, similar to the results of the random-effects model, suggesting that the meta-analyses were stable. Additionally, one-way sensitivity analysis (30) was also used to evaluate the stability of the meta-analysis. The statistical significance of any of the results was not altered by the omission of any single study, suggesting that the data in this meta-analysis were relatively stable and credible.

### Bias diagnostics

**Figure 5:**
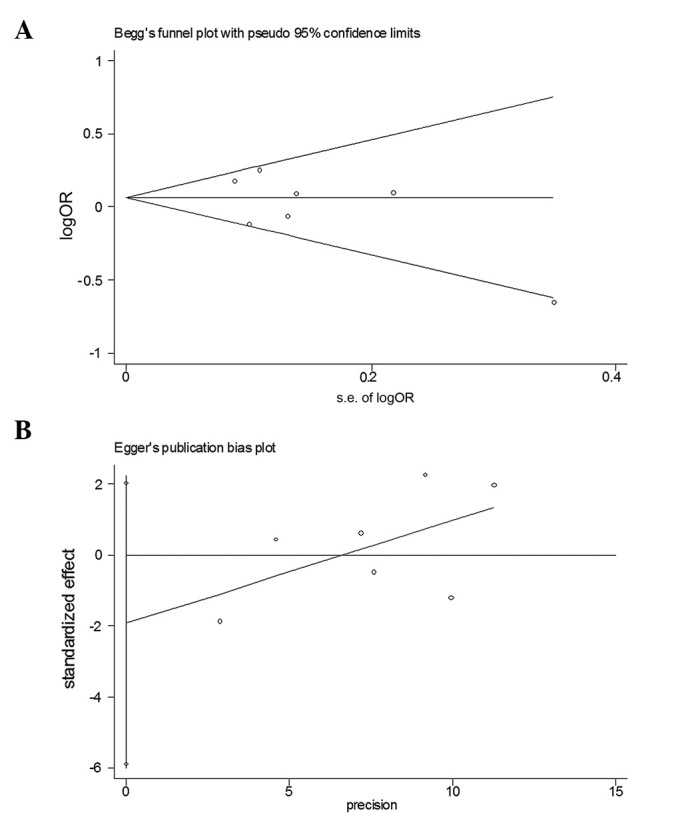
Publication bias tests. (A) Funnel plot; (B) Egger’s linear regression test. OR, odds ratio; s.e., standard error.

A funnel plot was created to assess possible publication biases (Fig. 5A). Egger’s linear regression test was then used to assess the symmetry of the plot (Fig. 5B). The data suggested that the funnel plot was symmetrical (t=−1.25, P>0.05), indicating that the results of the meta-analyses were relatively stable and that publication bias had little effect on the results.

## Discussion

In the present study, the overall data of the meta-analyses showed that the *NAT2* polymorphism may not have a significant association with colorectal adenoma risk. Nevertheless, in subgroup analysis, slow acetylator variants may modify the colorectal adenoma susceptibility of individuals who have a history of smoking.

Possible correlations of *NAT2* polymorphisms with cancer risk were evaluated by the meta-analyses. Previously, *NAT2* polymorphisms have been indicated to be associated with an increased susceptibility to prostate cancer and laryngeal cancer in Asian populations (31,32). However, meta-analyses with regard to lung and gastric cancer did not reveal any marked associations (33,34). Similarly, meta-analyses published in 2002, 2006 and 2012 failed to demonstrate a significant association between *NAT2* polymorphisms and colorectal cancer risk (35–37). In the present study, the overall data also failed to reveal a significant association of the *NAT2* variants with colorectal adenoma, in line with the meta-analyses concerning colorectal cancer.

A previous meta-analysis suggested that smoking affected the formation and aggressiveness of colon adenomas (38). Another meta-analysis suggested that smoking may interact with certain gene variants, such as the *NQO1* genetic variant (39), in the development of colorectal adenomas. Thus, we attempted to extract relevant data from the primary literature to conduct a subgroup analysis. The data showed that slow acetylator variants may be associated with an increased adenoma risk in individuals who have a history of smoking. The data indicate possible interactions between smoking with slow acetylator variants in the pathological mechanisms of colorectal adenoma. The underlying mechanisms by which *NAT2* polymorphisms affect colon adenomas in individuals with a history of smoking are not fully understood. The *NAT2* gene is located on chromosome 8p22, encodes a 290-amino acid protein (40) and catalyzes the detoxification and/or activation of aromatic and heterocyclic amine carcinogens by two pathways. This metabolic reaction may result in detoxification by N-acetylation or bioactivation by O-acetylation which is often proceeded by *CYP450* hydroxylation (41). Polymorphisms of *NAT2* may lead to differences in the rate of arylamine metabolism and consequently cancer risk (42). Individuals with a history of smoking and *NAT2* genetic variants who are exposed to cigarette toxins, such as nickel sulphate and benzo(b)fluoranthene, may have a reduced detoxification ability, thus resulting in an increased risk of colon adenoma as well as cancer. However, only two of the studies provided sufficient data concerning smoking status for subgroup analysis. Biases may be noted due to the limited number of selected studies and sample sizes. Therefore, these results should be interpreted with care.

Subgroup analyses of the source of the controls also failed to reveal significant associations in studies using hospital- or population-based controls. Since hospital-based controls may not always truly represent the general population, particularly when the controls have any disease-related conditions relevant to the studied genotypes, inherent selection biases may exist. Further well-designed studies, including population-based controls with strict matching criteria, are required to reduce such possible biases.

Inter-study heterogeneity was observed in this study. Thus, the random-effects model was used. Subgroup analyses were then conducted. As expected, the heterogeneities of the subgroups were reduced or removed. The data suggested that the source of the controls and smoking status may partly contribute to the heterogeneities. Additionally, other factors, including age, gender, ethnicity and the prevalence of lifestyle factors, may also generate heterogeneities.

Publication bias is an important factor that should be considered in a meta-analysis. In the present study, a funnel plot and Egger’s test were used to assess publication biases. The results indicated that publication biases exerted little effect on the overall results, suggesting that the meta-analysis was robust and credible.

Certain limitations may be included in this study. Firstly, in this meta-analysis, the majority of published studies and papers written in English were searched. However, it is possible that some related published or unpublished studies that may have met the inclusion criteria were missed. Thus, it is inevitable that publication biases may exist in the results. Secondly, all the studies concerned mixed ethnicities, with the exception of one study by Tiemersma (28) involving a Caucasian population. Therefore, subgroup analysis concerning ethnicity was not conducted and we were unable to assess the possible effects of ethnic variation on the results. Further studies conducted on separated ethnicities in various areas are required. Thirdly, subgroup analyses of age, gender, alochol use and meat consumption were not performed since insufficient information was available in the primary literature. Gene-gene and gene-environment interactions should also be considered in the further investigations.

In conclusion, although no significant associations of *NAT2* genetic variations with the overall risk of colorectal adenoma were revealed, statistically significant findings were noted in individuals with a history of smoking, suggesting that *NAT2* genetic variations may modify the colorectal adenoma risk of smokers. Further investigations are required to confirm this conclusion.

## Figures and Tables

**Table I:** Characteristics of the studies included in the meta-analysis.

First author (ref.)	Publication year	Cases (male/female)	Controls (male/female)	Type of controls	Age range (mean), years		Ethnicity	Country
					Cases	Controls		
Lang (24)	1994	41 (28/13)	205 (129/76)	205 volunteers (population-based)	36–84 (60.6)	20–80 (46.9)	Mixed	USA
Probst-Hensch (26)	1996	441 (280/161)	484 (326/158)	484 controls (age- and gender-matched; population-based)	50–74 (61.7)	50–74 (61.6)	Mixed	USA
Ishibe (23)	2002	146 (111/35)	228 (144/84)	228 controls (age- and gender-matched; hospital-based)	18–74 (58)	18–74 (59)	Mixed	USA
Tiemersma (28)	2004	431 (236/195)	433 (160/273)	433 controls (age- and gender-matched; hospital-based)	18–75 (58.8)	18–75 (50.4)	Caucasian	Netherlands
Moslehi (25)	2006	772 (535/237)	777 (536/241)	777 controls (gender- and ethnicity-matched; population-based)	55–74 (NA)	55–74 (NA)	Mixed	USA
Shin (27)	2008	557 (418/139)	1493 (1250/243)	1493 controls (hospital-based)	40–75 (59.6)	40–75 (57.2)	Mixed	USA
Wang (29)	2011	914 (550/364)	1185 (745/440)	1185 controls (age-, gender- and ethnicity-matched; hospital-based)	NA (61)	NA (62)	Mixed	USA

NA, not available.

**Table II:** Distribution of *NAT2* acetylator variants among colorectal adenoma cases and controls included in the meta-analysis.

First author	Year	Genotyping method	Cases		Controls	
			Rapid	Slow	Rapid	Slow
Lang	1994	Use of caffeine	25	16	92	113
Probst-Hensch	1996	AS-PCR	213	228	226	258
Ishibe	2002	PCR-RFLP	64	79	98	110
Tiemersma	2004	PCR-RFLP	168	259	179	253
Moslehi	2006	Taqman	272	413	317	376
Shin	2008	Taqman	243	311	609	880
Wang	2011	PCR-RFLP	449	457	631	539

NAT2, N-acetyltransferase 2; AS-PCR, allele-specific polymerase chain reaction; PCR-RFLP, polymerase chain reaction-restriction fragment length polymorphism.

**Table III:** Main results of the pooled data in the meta-analysis.

Characteristic	Slow vs. rapid acetylator variants			
	Number of cases/controls	OR	95% CI	P-value for Q-test
Overall	3197/4681	1.04	0.90–1.21	0.043
Smoking status				
Never smoked	387/481	0.92	0.53–1.58	0.051
Have smoked	665/561	1.31	1.04–1.64	0.795
Source of control				
Population-based	1167/1382	0.96	0.66–1.39	0.019
Hospital-based	2030/3299	1.05	0.90–1.23	0.172

OR, odds ratio; 95% CI, 95% confidence interval.
